# Neonatal hypoxia-ischemia in rat elicits a region-specific neurotrophic response in SVZ microglia

**DOI:** 10.1186/s12974-020-1706-y

**Published:** 2020-01-18

**Authors:** Urs Fisch, Catherine Brégère, Florian Geier, Laurie Chicha, Raphael Guzman

**Affiliations:** 1 Department of Neurology, University Hospital Basel, University Basel, Basel, Switzerland; 2 Brain ischemia and regeneration, Department of Biomedicine, University Hospital Basel, University Basel, Basel, Switzerland; 3 Bioinformatics Core Facility, Department of Biomedicine, University Basel, Basel, Switzerland; 4 Swiss Institute of Bioinformatics, Basel, Switzerland; 5 Department of Neurosurgery, University Hospital Basel, University Basel, Basel, Switzerland; 6 Faculty of Medicine, University Basel, Basel, Switzerland

**Keywords:** Microglia, Subventricular zone, Hypoxia-ischemia, Development, Neurogenesis, Rat

## Abstract

**Background:**

Recent findings describe microglia as modulators of neurogenesis in the subventricular zone (SVZ). SVZ microglia in the adult rat are thought to adopt a neurotrophic phenotype after ischemic stroke. Early postnatal microglia are endogenously activated and may therefore exhibit an increased sensitivity to neonatal hypoxia-ischemia (HI). The goal of this study was to investigate the impact of cortico-striatal HI on the microglial phenotype, function, and gene expression in the early postnatal SVZ.

**Methods:**

Postnatal day (P)7 rats underwent sham or right-hemispheric HI surgery. Microglia in the SVZ, the uninjured cortex, and corpus callosum were immunohistochemically analyzed at P10, P20, and P40. The transcriptome of microdissected SVZ and cortical microglia was analyzed at P10 and P20, and the effect of P10 SVZ microglia on neurosphere generation in vitro was studied.

**Results:**

The microglial response to HI was region-specific. In the SVZ, a microglial accumulation, prolonged activation and phagocytosis was noted that was not observed in the cortex and corpus callosum. The transcriptome of SVZ microglia and cortical microglia were distinct, and after HI, SVZ microglia concurrently upregulated pro- and anti-inflammatory as well as neurotrophic genes. In vitro, microglia isolated from the SVZ supported neurosphere generation in a concentration-dependent manner.

**Conclusions:**

Microglia are an inherent cellular component of the early postnatal SVZ and undergo developmental changes that are affected on many aspects by neonatal HI injury. Our results demonstrate that early postnatal SVZ microglia are sensitive to HI injury and display a long-lasting region-specific response including neurotrophic features.

## Background

Hypoxia-ischemic encephalopathy (HIE) is a neonatal brain injury most commonly caused by birth asphyxia. HIE occurs in 1–3 per 1000 live full-term births, but rises up to 40 per 1000 in preterm born with very low birth weight [1, 2]. Of affected infants, 25% develop persistent neurological impairments, including cerebral palsy, disorders of cognition and behavior, sensation, motricity, and epilepsy [3]. Currently, hypothermia is the standard of care in patients with moderate to severe HIE, and different therapeutic approaches are investigated in clinical trials [4, 5].

The rodent model of neonatal hypoxic-ischemic encephalopathy (HI) reflects the key pathomechanisms of HIE in term-born infants and has been shown to be a strong inducer of early postnatal SVZ neurogenesis [6–8]. Up to now, there are no clinical therapies available that specifically modulate this induced neurogenesis to amplify repair processes. Therefore, further research on HI-induced SVZ neurogenesis is needed.

Accumulating evidence indicates that microglial cells in the neurogenic niches bear an exclusive function as modulators of neural stem cell (NSC) and neural progenitor cell (NPC) proliferation and differentiation [9]. In the adult rat, SVZ microglia adopt a proneurogenic phenotype after ischemic stroke [10] and are therefore a potential therapeutic target to enhance regeneration after neonatal HI.

Microglia are the resident tissue macrophage of the central nervous system (CNS) and are essential to maintain homeostasis in health and to initiate immune responses in disease. Rodent microglia colonize the developing brain as early as E8.5 [11], before the CNS undergoes extensive developmental maturation. They play an active role in the CNS development by phagocytosis [12], synaptic pruning [13], regulation of axonal outgrowth, and positioning of interneurons [14] as well as trophic support of cortical neurons [15] and oligodendrocytes [16, 17]. Lately, the different stages of microglial development have been extensively characterized using genome-wide expression profiling. Up to eight different clusters of microglia appear to exist throughout development from E14.5 until late age, with the highest diversity being observed during embryonic and early postnatal development, when microglia are still differentiating [17–20].

Microglial diversity exists not only temporally during development, but also spatially in different brain regions [21], underscoring the necessity to study microglia in the context of their microenvironment. The knowledge about the effects of microglial sub-populations in the developing postnatal brain is still scarce. Within the first postnatal weeks, while “maturing”, SVZ microglia are highly activated, proliferative, and phagocytic; display an amoeboid shape; and express the lysosomal marker CD68 [16, 22]. In contrast, in the physiological adult brain, microglia do not express CD68 anymore and are thought to have a more surveilling role of their environment [23].

HI brain injury may cause diverse reactions by distinct, yet immature microglial populations in the developing early postnatal brain. We hypothesized that after HI, SVZ microglia adopt a specific phenotype which may in some aspects be supportive for HI-induced SVZ neurogenesis, similar to the adult SVZ microglia after ischemic stroke. Therefore, this study provides an in-depth characterization of the microglial phenotype in the early postnatal rat SVZ and the impact of neonatal HI on their development.

## Methods

### Hypoxia-ischemia neonatal rat model

**Fig. 1:**
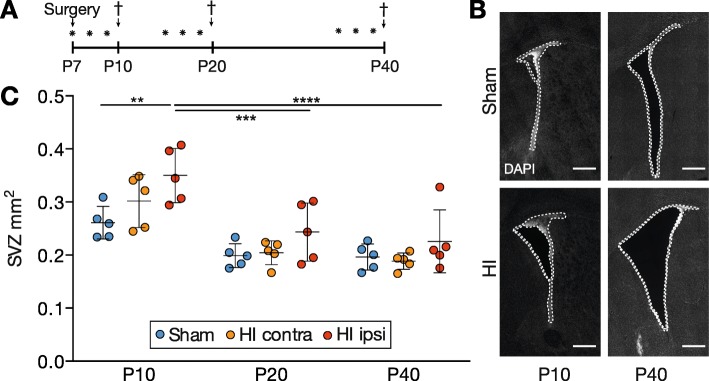
The SVZ temporarily expanded in size after neonatal HI. **a** Experimental timeline for the in vivo studies. Animals were subjected to sham or HI surgery at P7 and sacrificed at P10, P20, or P40. BrdU was injected for three consecutive days before sacrifice (asterisk). **b** Representative images of the ipsilateral SVZ after sham and HI surgery at P10 and P40. **c** Quantification of the SVZ size after sham and HI surgery. Individual data shown as dots, bars as mean with SD (error bar). Two-way ANOVA with Tukey post hoc test, **p < 0.01, ***p < 0.001, ****p < 0.0001 (Additional file 1: Table S3). Scale bar for **b**, 500 μm

**Fig. 2:**
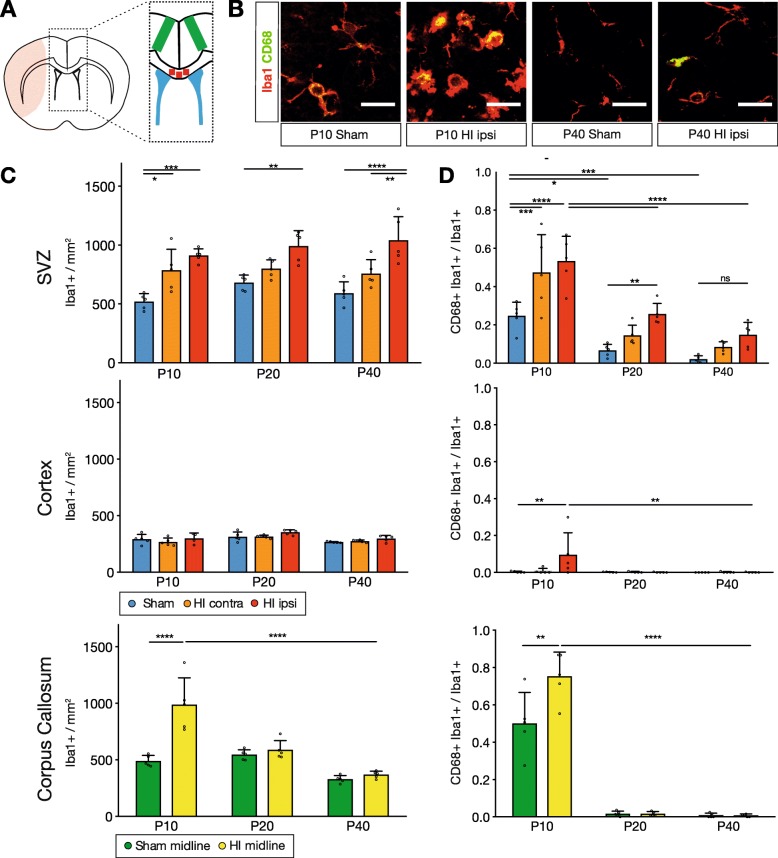
Microglia specifically accumulated early in the SVZ and displayed prolonged activation after HI. **a** Illustration of the analyzed regions in the HI-affected forebrain (pale red), including the SVZ (blue), the M2 supplementary motor cortex (green), and the midline corpus callosum (red). **b** Representative images of CD68+ Iba1+ activated microglia in the dorsolateral SVZ. **c** Microglial density in different brain regions. **d** Proportion of activated microglia in different brain regions. Individual data shown as dots, bars as mean with SD (error bar). Two-way ANOVA with Tukey post hoc test, ns = non-significant, *p < 0.05, **p < 0.01, ***p < 0.001, ****p < 0.0001 (Additional file 1: Table S3). Scale bar for **b**, 20 μm

All animal experiments were approved by the local veterinarian authorities and complied with the Swiss animal welfare guidelines. Sprague Dawley rats were bred in-house, and the day of birth was considered as P0. P7 rat neonates were randomly assigned to sham or HI surgery, with a balanced representation of both sexes among the two surgery groups. One hour prior to surgery, animals were injected intraperitoneally with buprenorphine (0.03 mg/kg body weight). Anesthesia was induced with 5% isoflurane and maintained with 3% during the surgery. The HI surgery consisted of a modification of the Rice-Vannucci model: the right common carotid artery was exposed and temporarily clipped with a Sugita 4-mm aneurysm mini clip (Mizuho) and the skin incision temporarily sutured. Animals recovered for 10 min on a heating pad and were then placed for 40 min into a hypoxic chamber constantly flushed with 8% oxygen/92% nitrogen gas mixture (2 L/min) that was immersed in a temperature-controlled water bath to maintain an air temperature of 37.5 °C. During hypoxia, epileptic seizures were observed in some animals as previously reported [24] but were not an exclusion criterion for further analyses. Animals then recovered for 10 min on a heating pad and were briefly anesthetized with isoflurane for clip removal and permanent skin suture. A typical neonatal HI injury pattern consists of an ipsilateral cortico-striatal injury sparing out the medial regions of the hemisphere (Fig. 2a) and a chronic ventriculomegaly due to brain volume loss (Fig. 1b). The sham surgery consisted of artery exposure without temporary occlusion and without hypoxia exposure. A subgroup of sham-assigned animals underwent a “hypoxia only” procedure with sham surgery followed by hypoxia exposure as described above without temporary carotid artery clipping.

### BrdU administration and brain collection for stainings

Animals received daily single intraperitoneal bromodeoxyuridine (BrdU) injections (100 mg/kg body weight, Sigma) for three consecutive days after surgery and before sacrifice (Fig. 1a). Animals were then sacrificed at P10, P20, or P40 to reflect acute, subacute, and chronic stages after HI (*n* = 5 sham and *n* = 5 HI per time point, three animals at P10 for “hypoxia only”). Transcardiac perfusion with 0.9% saline under deep anesthesia, followed by 4% paraformaldehyde (PFA) in 0.1 M phosphate buffer pH 7.4 (PB) was performed. Brains were post-fixed in 4% PFA in PB for 48 h at 4 °C, cryoprotected in consecutive 15% and 30% sucrose solutions, embedded in OCT (Leica Biosystems), and cryosectioned. Coronal free-floating sections (30 μm) were stored at − 20 °C in a cryoprotectant solution (30% ethylene glycol, 30% sucrose in PB) until staining.

### Cresyl violet staining and animal selection for histological studies

Coronal brain sections (interval 180 μm) were mounted on slides (Superfrost plus, Menzel), stained with 0.1% cresyl violet acetate (Sigma), and scanned (Nikon Eclipse TI-E microscope). Brain sections including the anterior SVZ and 0.40 rostral and − 0.20 caudal to bregma in P10 rats [25] (corresponding anatomical sections for P20 and P40 rats, respectively) were investigated. Due to the significant variability in HI injury size in the Rice-Vannucci model of neonatal HIE, two investigators (UF, CB) independently assessed the HI injury size using ImageJ software (version 2.00rc.43/1.50e) and their results were averaged. The size of HI injury was calculated by subtracting the intact area in the right, from here on defined as the ipsilateral hemisphere from the total area of the left, contralateral hemisphere in 3 serial cresyl violet-stained sections as previously described [6]. Animals with no apparent or extensive HI injury that affected the SVZ were excluded in order to compare relatively homogenous groups. Thus, per time point, *n* = 5 sham animals and *n* = 5 HI with mild to moderate injury severity were selected (total of 30 with 17 females, 7 excluded), and *n* = 3 (2 females) P10 “hypoxia only” (Additional file 1: Table S1).

### Immunostaining

For immunohistochemistry, brain sections were washed in Tris-buffered saline (TBS), incubated in blocking buffer (TBS with 2% fish gelatine, 0.3% Triton X-100, Sigma) for 1 h at room temperature (RT), then incubated with primary antibodies (Table 1) overnight at 4 °C in blocking buffer, washed repetitively with TBS, and incubated with the corresponding species-specific Alexa-Fluor (488, 546, 555, 647)-conjugated donkey or goat (H+L) secondary antibodies (Thermo Fisher) in blocking buffer (1:2000) for 1 h at RT. Sections were counterstained with 0.5 μg/ml DAPI (Sigma) before mounting on superfrost slides with ProLong Gold (Thermo Fisher). For BrdU labeling, sections were pretreated with 2 N HCl for 30 min at RT before the blocking step. For proliferating nuclear cell antigen (PCNA) labeling, antigen retrieval was performed for 5 min at 80 °C in target retrieval solution (Dako) before blocking. For immunocytochemistry (Table 1), cell culture wells were fixed for 15 min in 4% PFA at RT. The immunofluorescence steps were identical to those described above.

**Table 1:** Primary antibodies used for stainings

Host/primary antibody	Producer	Concentration for histology	Concentration for cytology
Mouse/anti-BrdU	BD Bioscience	1:200	
Mouse/anti-CD68	R&D Systems	1:2000	
Guinea pig/anti-DCX	Merck Millipore		1:2000
Rabbit/anti-Iba1	Wako	1:2000	1:1000
Goat/anti-Iba1	Abcam	1:1000	
Rabbit/anti-Ki67	Abcam	1:1000	
Mouse/anti-Nestin	Millipore		1:500
Rabbit/anti-PAX6	Biolegend	1:1000	
Mouse/anti-PCNA	DAKO	1:700	

### Brain section image acquisition and analysis

The 20x wide-field immunofluorescence images from brain sections were acquired on an Eclipse TI-E microscope (Nikon), or 40x confocal immunofluorescence image stacks (330 × 330 μm, 11 μm depths, step size 0.5 μm) on a CSU-W1 microscope (Visitron Systems). Representative microglial activation images were acquired on a 40x LSM 710 confocal microscope (Zeiss). All quantitative analyses were conducted in three brain sections (interval 180 μm), and mean values were calculated per animal. Image acquisition settings were identical for all stainings.

#### SVZ area and microglial quantification

Quantifications of SVZ area and microglial morphology, density, and activation were performed manually with ImageJ from wide-field images. The SVZ area was defined by its DAPI+ cellular density (Fig. 1b). The microglial cell morphology was classified into three categories: (i) amoeboid with roundish shape without processes, (ii) intermediate with irregular shape and short processes, and (iii) ramified with processes extending more than twofold the diameter of the cell body (Fig. 4 A1–A3). The microglial density was calculated as the number of ionized calcium-binding adapter molecule 1 (Iba1)+ cells per area in three brain regions: (i) the SVZ, (ii) a 1600 × 200 μm rectangle including layers I–VI of the M2 supplementary motor cortex (CX), and (iii) in the sum of three 150-μm squares which were symmetrically distributed in the midline corpus callosum (CC) (Fig. 2a). Microglial activation, here defined by expression of CD68 [16], was calculated as the number of CD68+ Iba1+ cells per total number of Iba1+ cells for each region. The analysis was conducted in five animals per surgery and time point.

#### Quantification of cell proliferation and microglial ball-and-chain buds

Paired box 6 protein (PAX6)+ PCNA+ cells in the most medial part of the dorsolateral SVZ were manually counted in a confocal microscopy stack of 50 μm × 50 μm × 10 μm depth using ImageJ. Only cell nuclei being fully included within the stack were counted. Microglial proliferation (number of BrdU+ Iba1+ or Ki67+ Iba1+ cells per total number of Iba1+ cells) and ball-and-chain buds (number of ball-and-chain-shaped pouches per total number of Iba1+ cells) in the dorsolateral SVZ or CX were manually quantified in 3D-reconstructed confocal stack images with Imaris software (ver. 7.6.5, Bitplane). Ball-and-chain buds were defined as spherically shaped extensions at the terminal branch of microglial processes. Analysis was conducted in three animals per surgery and time point.

### Microglial isolation and RNA purification

Animals underwent sham or HI surgery at P7, and microglia were isolated from the SVZ and CX at P10 or P20 for subsequent transcriptome analysis (*n* = 6 animals per surgery and time point, total of 24 with 11 females; 38 excluded [Additional file 1: Table S1]). Animals were deeply anesthetized and perfused with ice-cold 0.9% saline. Brains were extracted and kept in ice-cold Hank’s balanced salt solution (HBSS) (Sigma) for the following sterile procedures. The region of the anterior SVZ was coronally cut with razor blades in 2-mm-thick sections and two sections selected. Sections from HI animals were further processed only if the HI injury severity was mild to moderate without affection of the ipsilateral SVZ or the adjacent medial striatum and corpus callosum (microscopic assessment during preparation). From individual HI animals, the ipsi-dorsolateral SVZ and the contralateral CX were microdissected under a dissection microscope (Leica) and separately collected. From individual sham animals, SVZ or CX from both hemispheres were microdissected. The individual tissue samples were processed at 4 °C. They were washed (centrifugation at 300*g* for 5 min, followed by aspiration of the supernatant), dissociated with the papain neural dissociation kit (Miltenyi), filtered through a 40-μm strainer, washed again, and magnetically labeled with mouse anti-rat CD11b microbeads (1:200) (Miltenyi) for 20 min. CD11b+ cells were ferromagnetically isolated using MS columns (Miltenyi) following the manufacturer’s instructions. The purity of CD11b+ sorted cells was tested by flow cytometry (see below). Due to the very small tissue samples and consecutively low cell yield, simultaneous flow cytometry analysis and RNA collection from individual tissue samples were not possible. Isolated CD11b+ cells were immediately processed with the Arcturus PicoPure RNA isolation kit (Thermo Fisher Scientific) including DNase treatment according to the manufacturer’s instructions and stored at − 80 °C after extraction buffer treatment. RNA isolation was then completed batch-wise. RNA integrity and concentration from CD11+ isolated cells were measured with the RNA 6000 Pico Kit (Agilent) on the 2100 Bioanalyzer (Agilent).

### Microglial transcriptome analysis

Sample preparation for microarray processing was performed externally at Life & Brain GmbH, Bonn, Germany. In brief, 500 pg total RNA per sample was reverse transcribed into cDNA using the GeneChip WT Pico Assay Kit (Affymetrix) in a two-step process according to the manufacturer’s instructions. The cDNA was subsequently fragmented, labeled, and hybridized to the GeneChip Rat Transcriptome Array 1.0 (Affymetrix). After staining, scanning was performed on a GeneChip Scanner 3000 (Affymetrix).

The raw microarray data was normalized using the R/Bioconductor package oligo (version 1.38.0). In brief, CEL files were read-in and subsequently normalized using the function rma. Probe sets were annotated with the affycoretools package (version 1.46.4). Entrez IDs with multiple probe sets were filtered for the probe set with the highest variance. One sample was identified as an outlier, and the animal (P20 sham SVZ) was removed from further analysis. The limma package (version 3.30.7) was used for differential gene expression analysis. One set of contrasts was defined to evaluate the surgery differences at both time points. *P* values were adjusted for multiple testing using the Benjamini and Hochberg false discovery rate (FDR), and genes with a FDR < 0.05 were considered as significant. Published microarray data sets were used to define M1 and M2 polarization markers by selecting the 15 most differentially expressed genes (FDR < 0.05) of lipopolysaccharide (LPS) or IL-4 stimulated microglia [26]. Gene sets defined in the Kyoto Encyclopedia of Genes and Genomes (KEGG) (version downloaded on 9 February 2017) were tested for differential enrichment using the limma function kegga. Principal component analysis and pathway-enrichment graphs were generated using R base graphics while heat maps for specified gene sets were based on the ComplexHeatmap package (version 1.12.0). Gene enrichment analysis for Gene Ontology (GO) terms for differentially expressed gene sets of SVZ and CX microglia were performed with the Database for Annotation, Visualization and Integrated Discovery (DAVID) v6.7.

### Quantitative real-time PCR

The microarray results were validated by quantitative real time-polymerase chain reaction (qPCR) from pooled RNA samples from isolated SVZ microglia as described above that were not included in the microarray analysis (500 pg per sample, *n* = 2 animals for sham, *n* = 2 for HI per time point, respectively [Additional file 1: Table S1]). Due to the low RNA amount (1 ng) per pooled sample group, we chose Igf-1 expression levels for validation. Pooled RNA was reverse-transcribed with the QuantiTect Reverse Transcription Kit (Qiagen), and qPCR was performed in triplicates using Fast SYBR Green MasterMix (Roche) on a LightCycler 480 (Roche). Primers for Igf-1 and β-actin were commercially acquired (QuantiTect, Qiagen). All samples were analyzed concurrently in one experiment. Runs were normalized to the housekeeping gene β-actin by measuring ΔCT. The 2^−ΔΔCT^ method was used for the calculation of the Igf-1 fold change (FC) of the HI vs. sham group per time point.

### Primary neurosphere generation from SVZ tissue and microglial depletion

Animals underwent sham or HI surgery at P7 (3 independent experiments with *n* = 3 sham animals and *n* = 6 HI per experiment, total of 27 with 15 females; 4 excluded [Additional file 1: Table S1]) and were deeply anesthetized and decapitated at P10. Rat heads were immersed in 70% ethanol for 30 s and kept in ice-cold sterile HBSS until sterile dissection. Coronal sections of 6 mm thickness of the anterior SVZ were prepared as described above. Sections from HI animals were further processed only if the HI injury severity was mild to moderate without affection of the ipsilateral SVZ or the adjacent medial striatum and corpus callosum (microscopic assessment during preparation). Rectangular tissue blocks including the whole ipsi- or contralateral SVZ and the adjacent medial striatum were isolated. From HI animals, ipsi- and contralateral tissue blocks were pooled separately, whereas from sham animals, all SVZ tissue blocks were pooled per experiment, resulting in three tissue groups: (i) ipsilateral HI, (ii) contralateral HI, and (iii) sham (Fig. 6 A1). The pooled tissue samples were washed, dissociated with the papain neuronal dissociation kit (Miltenyi), and filtered through a 70-μm strainer. The cell suspension was washed and counted (TC20 counter, Bio-Rad). Dissociated cells were seeded at a density of 0.3 Mio in uncoated 24-well plates (Corning). Cells were incubated in 500 μl neuronal expansion medium (DMEM/F12 1:1, Gibco; with N2 supplements [human apo-Transferrin, 100 mg/l; insulin, 25 mg/l; putrescine, 100 μM; sodium selenite, 30 nM; Sigma]; penicillin/streptomycin, 100 μg/ml, Gibco; glutaMAX, 2 mM, Gibco). The cell culture plates were incubated at 37 °C in 5% CO_2_. The medium was completely replaced after 2 h and plates treated in three different conditions: (i) addition of microglia-targeting mouse anti-CD11b saporin-conjugated antibodies (anti-CD11b SAP, 0.35 μg/ml) (Advanced Targeting Systems), (ii) unspecific mouse IgG saporin-conjugated antibodies (IgG SAP, 0.35 μg/ml) (Advanced Targeting Systems), or (iii) no addition of antibodies (control) (Fig. 6 A2). Cell cultures were supplemented daily with recombinant human fibroblast growth factor (20 ng/ml, R&D Systems) and recombinant human epidermal growth factor (20 ng/ml, Peprotech). Half of the medium was replaced at 3 days in culture (DIC). Neurospheres were analyzed after 6 DIC.

### Cell culture image acquisition and analysis

Two 2.5x wide-field images per well were acquired on an Axiovert 200 microscope (Zeiss), representing 25% of the total well surface. Number and area of individual neurospheres with a diameter > 45 μm were automatically counted using ImageJ. For each experimental condition, the data from three wells were averaged and extrapolated to calculate neurosphere numbers per well. Qualitative confocal 40x fluorescence images were acquired from immunocytochemically stained wells.

### Flow cytometry

Dissociated samples were washed and stained with mouse anti-CD11b-FITC (1:200) (Bio-Rad) and mouse anti-CD45-PE (1:200) (Bio-Rad) in buffer solution (phosphate-buffered saline pH 7.4, 0.5% bovine serum albumin, 2 mM ethylenediaminetetraacetic acid [EDTA]) at 4 °C for 20 min, followed by washing and processed on an Accuri flow cytometer (BD Bioscience). All data were analyzed with FlowJo software (version 10.2).

### Statistical analysis

All data sets except the microarray data were analyzed in Prism software (GraphPad, version 6). All data are presented as mean ± standard deviation (SD) if not stated otherwise. Statistical analysis was performed with two-way ANOVA with Tukey post hoc test for age or surgery, or one-way ANOVA with Holm-Sidak post hoc test. *P* values < 0.05 were considered as significant. Correlations were computed with two-tailed Pearson correlation coefficients or nonparametric Spearman correlation. Countings for the SVZ size, microglial density, and proportion of activated microglia from ipsi- and contralateral sham hemispheres did not show any significant differences (Additional file 1: Figure S1). Thus, only ipsilateral (right-sided) sham data were used for statistical analysis of histological data.

## Results

### The SVZ transiently enlarges after HI

Neonatal HI was shown to induce a temporary enlargement of the SVZ and an increase in NSC/NPC proliferation [27, 28]. We measured the area of the total SVZ in sham and HI animals at P10, P20, or P40, reflecting acute, subacute, and chronic stages of HI injury (Fig. 1a, b). Due to the significant variability in HI injury severity in the Rice-Vannucci model of neonatal HIE, HI injury size was independently assessed by two investigators (Pearson coefficient *r* = 0.96, 95% confidence interval 0.91–0.98, *p* < 0.0001, *n* = 34) and animals selected with mild to moderate HI injury severity (median 49% hemispheric injury size [interquartile range 35–60%]) without histological signs of HI damage to the anterior SVZ.

HI induced an enlargement of the SVZ at P10 that was most prominent in the ipsilateral HI SVZ as compared to the sham SVZ (0.35 [mean] ± 0.05 [SD] vs. 0.26 ± 0.03 mm^2^, *p <* 0.004) (Fig. 1c). This enlargement was transient, and at P20 and P40, the HI ipsilateral SVZ normalized again to the size of sham SVZ (for P40, 0.23 ± 0.06 vs. 0.20 ± 0.02 mm^2^), whereas ventriculomegaly persisted (Fig. 1b). Overall, the size of the SVZ markedly decreased with age with an early transient enlargement in the ipsilateral hemisphere of HI animals.

### Microglia in the SVZ specifically accumulate early and remain activated after HI

After ischemic stroke in the adult rat, microglia in the ipsilateral SVZ become activated and accumulate over weeks [10]. Early postnatal microglia are not yet fully mature and are endogenously activated in the SVZ [16–20, 22], a developmental feature which may affect their response to injury. We therefore analyzed the impact of HI on microglia in the SVZ, CC, and CX by quantifying their density and the proportion of activated microglia (Fig. 2a, b).

After HI, microglia in the ipsilateral SVZ significantly accumulated at P10 (ipsilateral HI SVZ, 911 ± 57 vs. sham SVZ, 519 ± 67 Iba1+/mm2, *p* < 0.0001). This early increase in microglial density in HI SVZ remained constant until P40 when compared to sham SVZ (ipsilateral HI SVZ 1041 ± 200 vs. sham SVZ 590 ± 66, *p* < 0.0001) (Fig. 2c). Likewise, HI led to a striking increase in the proportion of activated microglia in the ipsilateral SVZ at P10 (ipsilateral HI SVZ 0.53 ± 0.13 vs. sham SVZ 0.25 ± 0.07 CD68+ Iba1+/Iba1+, *p* < 0.0001) and P20 (ipsilateral HI SVZ 0.26 ± 0.06 vs. sham SVZ 0.06 ± 0.03, *p* < 0.006), while overall, the proportion of activated microglia decreased in both groups with age (Fig. 2d). However, with increasing age, a substantial reduction of activated microglia was observed in both HI ipsilateral SVZ (P10, 0.53 ± 0.13 vs. P40, 0.08 ± 0.03, *p* < 0.0001) and sham SVZ (P10, 0.25 ± 0.07 vs. P40, 0.02 ± 0.02, *p ≤* 0.001). Thus, microglia in the ipsilateral SVZ responded to HI with early accumulation and prolonged activation, while during the same period, the number of PCNA+ PAX6+ NPCs remained unchanged (Additional file 1: Figure S2).

We next investigated whether these findings were specific to the SVZ and quantified the same parameters in the adjacent CX and CC (Fig. 2a). In the CX, the microglial density of HI vs. sham remained unchanged from P10 (ipsilateral HI CX, 299 ± 47 vs. sham CX, 310 ± 49 Iba1+/mm^2^) until P40 (ipsilateral HI CX, 297 ± 28 vs. sham CX, 266 ± 6), while in the CC, it transiently increased at P10 (HI CC, 989 ± 238 vs. sham CC, 490 ± 49, *p* < 0.0001) but thereafter returned to sham levels until P40 (HI CC, 367 ± 31 vs. sham CC, 328 ± 32) (Fig. 2c). At P10, HI led to an increased proportion of activated microglia in the CX (ipsilateral HI CX, 0.1 ± 0.12 vs. sham CX, 0.003 ± 0.004 CD68+ Iba1+/Iba1+, *p* < 0.003) and in the CC (HI CC, 0.75 ± 0.13 vs. sham CC, 0.5 ± 0.17, *p* < 0.01); nevertheless, by P20, the proportion of activated microglia was equally reduced in all groups (Fig. 2d).

In summary, microglia in regions other than the SVZ were activated for a shorter time as compared to SVZ microglia and did not demonstrate a sustained accumulation. Interestingly, the SVZ contralateral to the ischemic hemisphere showed similar results as the ipsilateral HI SVZ, although less pronounced (Figs. 1c and 2c, d). To verify that the findings in the contralateral SVZ were due to the combination of global hypoxia and unilateral ischemia and not to hypoxia alone, rat neonates were subjected to sham surgery followed by exposure to hypoxia (*n* = 3 “hypoxia only” animals). The results in this group were similar to those measured in sham animals, underscoring the specificity of the microglial response to the HI injury in the ipsilateral and contralateral SVZ (Additional file 1: Figure S3).

### SVZ microglia proliferate early after HI

**Fig. 3:**
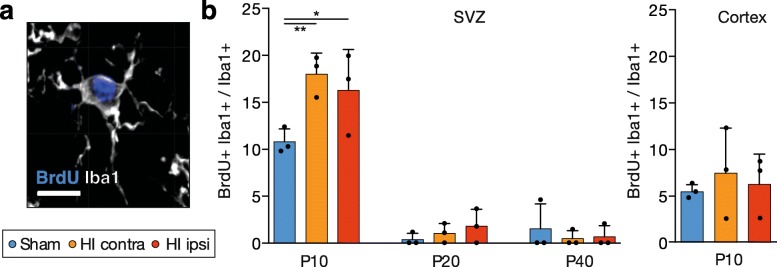
HI induced early proliferation of SVZ microglia. **a** Representative image of a BrdU+ microglia. **b** The proportions of BrdU+ Iba1+ proliferating microglia among total numbers of microglia in the dorsolateral SVZ and cortex. Individual data shown as dots, bars as mean with SD (error bar). Two-way ANOVA with Tukey post hoc test, *p < 0.05, **p < 0.01 (Additional file 1: Table S3). Scale bar for **a**, 10 μm

We then asked if HI-induced microglial accumulation in the SVZ was due to local proliferation. BrdU was administered for three consecutive days before sacrifice, and proliferating BrdU+ microglia were quantified in the dorsolateral SVZ (Fig. 3a). The proportion of proliferating microglia in sham animals was higher at P10 than afterwards. HI led to a significant increase in microglial proliferation at P10 in the ipsilateral and contralateral SVZ (ipsilateral HI, 0.16 ± 0.04 vs. sham, 0.11 ± 0.01 BrdU+ Iba1+/Iba1+, *p* < 0.014) (Fig. 3b). The analysis of Ki67+ Iba1+ proliferating cells at P10 depicted similar, but non-significant trends (Additional file 1: Figure S4), suggesting that the HI-induced proliferation mainly occurred before P10. In comparison, microglial proliferation in the CX was noticeably lower and remained unaffected by HI at P10 (ipsilateral HI, 0.06 ± 0.03 vs. sham, 0.05 ± 0.01) and was practically inexistent thereafter (data not shown). Hence, microglia proliferated early after birth and more pronounced in the SVZ after HI.

### HI temporarily increases the proportion of amoeboid SVZ microglia

**Fig. 4:**
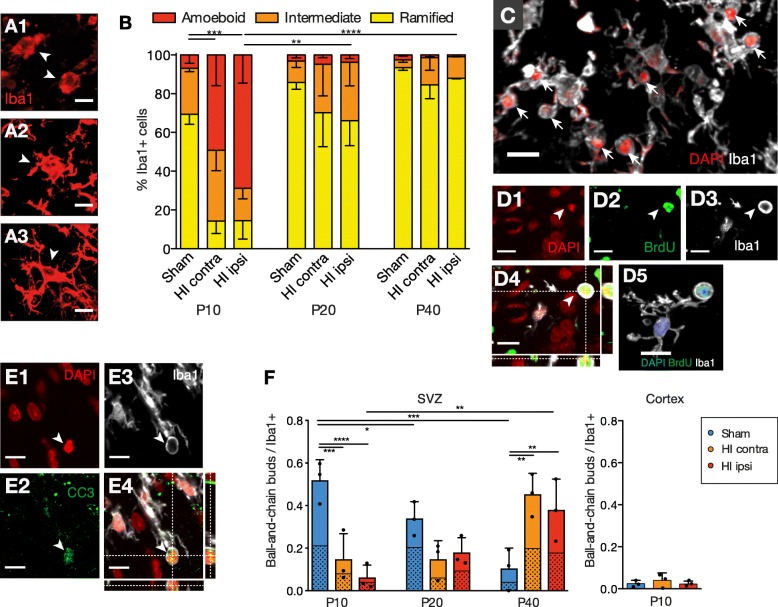
HI affects microglial morphology and phagocytosis. **A1**, **A2**, **A3** Microglia were classified upon their morphology to **A1** amoeboid, **A2** intermediate, or **A3** ramified (arrows). **B** Proportions of Iba1+ microglia in the dorsolateral SVZ according to their morphology. Statistical analysis for ramified microglia. Mean with standard error of the mean (error bar). **C** 3D-reconstructed representative image highlighting the content of ball-and-chain microglial buds (arrows) that consist of pyknotic DAPI+ cells. **D1**, **D2**, **D3**, **D4**, **D5** Representative images of a pyknotic BrdU+ cell engulfed by a microglial bud (arrow) with **D1** DAPI, **D2** BrdU, **D3** Iba1 stainings, **D4** the correspondent composite stack image, and **D5** a 3D-reconstructed image of the whole microglia cell. **E1**, **E2**, **E3**, **E4** Representative images of a pyknotic CC3+ cell engulfed by a microglial bud (arrow) with **E1** DAPI, **E2** CC3, **E3** Iba1 stainings, and **E4** the correspondent composite stack image. **F** Number of ball-and-chain buds per Iba1+ microglia in the dorsolateral SVZ and the cortex. The polka dot-filled bar area indicates the mean number of BrdU+ containing ball-and-chain buds. Individual data shown as dots, bars as mean with SD (error bar). Two-way ANOVA with Tukey post hoc test, *p < 0.05, **p < 0.01, ***p < 0.001, ****p < 0.001 (Additional file 1: Table S3). Scale bar for **A**, **C**, **D**, **E**, 10 μm

Microglia in the dorsolateral SVZ were classified based on their morphology into amoeboid, intermediate, and ramified phenotypes (Fig. 4 A1–A3). An amoeboid morphology is associated with increased activation, proliferation, and phagocytosis [23]. In both sham and HI animals, the proportion of amoeboid microglia decreased with age (Fig. 4B). At P10, HI caused a striking increase in the percentage of amoeboid microglia in both ipsilateral and contralateral SVZ (ipsilateral HI, 68.9 ± 25.2%; contralateral HI, 49.2 ± 27.6%; sham, 6.8 ± 7.4%, *p ≤* 0.01). However, this difference was no longer present afterwards (P40, ipsilateral HI, 0.9 ± 0.3%; contralateral HI, 1.4 ± 1.6%; sham, 2.6 ± 1.1%). In opposite, the proportion of ramified microglia was significantly lower at P10 after HI compared to sham (ipsilateral HI, 14.5 ± 16.5%; contralateral HI, 14.3 ± 11.0%; sham, 69.4 ± 9.1%, *p ≤* 0.001), but no longer at P40 (ipsilateral HI, 87.9 ± 0.6%; contralateral HI, 84.6 ± 12.4%; sham, 93.4 ± 2.3%). In comparison, CX microglia in HI and sham animals remained ramified from P10 until 40 (data not shown). Thus, the specific endogenous activation of microglia in the early postnatal SVZ was markedly amplified by HI.

### Increased number of ball-and-chain buds among SVZ microglia after HI

Phagocytosis is a key feature of microglia in the developing brain and maintains homeostasis in the adult neurogenic niches mediated through ball-and-chain-shaped pouches that engulf and phagocyte pyknotic cells [29, 30]. We therefore assessed microglial ball-and-chain buds in the dorsolateral SVZ which are best detectable in ramified microglia (Fig. 4C, D1–D5, E1–E4) [29].

In the sham SVZ, the number of ball-and-chain buds among Iba1+ cells were most prominent at P10 and continuously decreased thereafter (P10, 0.52 ± 0.1 vs. P40, 0.01 ± 0.1, *p* ≤ 0.0002) (Fig. 4F). This developmental course was reversed in HI animals (ipsilateral HI SVZ; P10, 0.06 ± 0.06 vs. P40, 0.38 ± 0.15, *p* < 0.003). Thus, at P40, microglia in the SVZ from HI animals showed significantly more ball-and-chain buds than in that from sham animals (ipsilateral SVZ, 0.38 ± 0.15 vs. sham SVZ, 0.01 ± 0.1, *p* < 0.009). Approximately half of the ball-and-chain buds contained BrdU+ nuclei (Fig. 4 D1–D5, F), indicating that the engulfed cells had recently proliferated. Microglial ball-and-chain buds in the CX were rare at P10 (Fig. 4F) and undetectable afterwards (data not shown). Engulfed cells rarely stained for the apoptotic marker cleaved caspase 3 (CC3) (Fig. 4E1–E4). They remained negative for NSC and NPC markers (sex determining region Y-box 2 [SOX2], doublecortin [DCX], PAX6, polysialylated-neural cell adhesion molecule [PSA-NCAM], data not shown) which may be due to rapid protein degradation [29], or alternatively other cells were engulfed. In conclusion, among ramified microglia, ball-and-chain buds were most prominent early at P10 in sham animals and late at P40 in HI animals.

### SVZ microglia upregulate pro- and anti-inflammatory and neurotrophic genes after HI

**Fig. 5:**
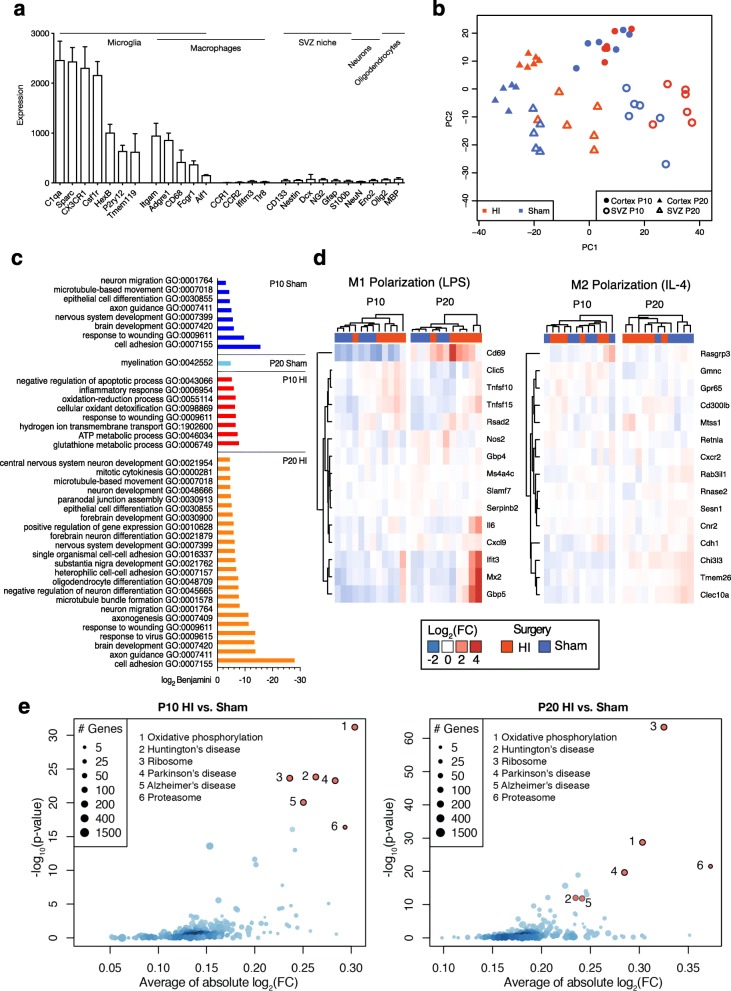
SVZ microglia upregulate pro- and anti-inflammatory and neurotrophic genes after HI. **a** Expression of specific genes for different cell types in microdissected CD11b+ microglia from the SVZ and cortex. Bars as mean with SD (error bar). **b** Principal component analysis of all samples. **c** Gene annotation enrichment analysis for significantly and > 1.5-fold upregulated genes in SVZ microglia compared to cortex microglia. **d** Heat maps for SVZ microglial expression of M1 (LPS)-polarized or M2 (IL4)-polarized genes. **e** KEGG pathways enrichment in SVZ microglia

We analyzed the transcriptome of Cd11b+ cells microdissected and purified from the SVZ or CX of individual sham and HI animals at P10 and P20. Flow cytometry analysis demonstrated a concentration of > 95% CD11b+ CD45+ cells after isolation (Additional file 1: Figure S5). Total RNA was extracted from these cells (RIN ≥ 7.2 [mean 9.6]), and a total of 500 pg RNA per sample was used for microarray experiments. The microarray analysis revealed high expression of microglia-specific transcripts, including C1qa, Cx3cr1, P2ry12, and Tmem119 among all samples, whereas the macrophage-specific gene expression was low (Fig. 5a). The principal component analysis demonstrated a grouping of most samples of the same condition with age and anatomical origin being the main components before surgery (Fig. 5b). The microarray results were validated by qPCR of additional SVZ samples that were not used in the microarray (Additional file 1: Figure S6).

The difference in gene expression of SVZ and CX microglia was age-dependent. In P10 sham animals, microglia in the SVZ significantly (FDR < 0.05) upregulated 2319 and downregulated 1362 genes compared to microglia in the CX. In contrast, at P20, they were only 151 significantly upregulated and 33 downregulated genes. Some upregulated genes in SVZ microglia were consistent with increasing age, including Axl, Clec7a, Fabp7, Lrrc74b, Olr1, Olr1366, Rab7b, Sulf2, and Zcchc18, while other gene upregulations were only found at a specific time point, such as at Cd8b, Dcx, Gpnmb, Gpr165, Hsdl2, Igf1, Ms4a6bl, Pdk4, and Spp1 at P10.

We next performed gene-annotation enrichment analysis for significantly and minimally 1.5-fold upregulated genes in SVZ microglia in comparison with CX microglia (Fig. 5c). At P10, the resulting GO terms in sham microglia were associated with cell motility, nervous system development, and axonal guidance, in difference to metabolic and inflammatory processes and negative regulation of apoptosis in HI animals. At P20, in sham microglia, only the GO term myelination was present, whereas in HI microglia, the GO terms associated with cell motility, nervous system development, and negative regulation of neuronal regulation shared some resemblance to those found in sham microglia at P10.

Thus, the difference of the SVZ and CX microglial gene expression underscored the dynamics and region-specificity of microglial development. The gene-annotation enrichment analysis indicated a long-term impact of HI onto SVZ microglial gene expression and potential functional effects.

We then analyzed in further detail gene expression differences in the SVZ. HI microglia significantly upregulated 1807 and downregulated 389 genes compared to sham at P10, and upregulated 2642 and 7288 downregulated genes at P20, respectively. Overall, the FC of significantly differentially expressed genes in the SVZ was modest. In HI compared to sham microglia, 19 genes were more than 2-fold upregulated and 3 downregulated at P10, and 25 upregulated and none downregulated at P20. The 25 most upregulated genes are listed in Table 2 for P10, and Table 3 for P20, respectively. Among those genes, we noticed a concurrent expression of genes associated with proinflammatory (RasGef1b, TNFsf9, Rab32, Stat1, Ifi27, Pdpn) and anti-inflammatory (Anxa2, CD206, Gpnmb, Clec7, miR-146a) effects as well as genes with neurotrophic effects such as Igf-1 [15] and C3 [31]. Thus, the transcriptome of SVZ microglia after HI shows a complex pattern of concurrent upregulation of pro- and anti-inflammatory as well as neurotrophic genes.

**Table 2:** Upregulated genes in SVZ microglia after HI at P10

Gene symbol	Gene name	EntrezID	FC HI vs. sham
Emb	Embigin	114511	4.59
LOC100911403	Membrane-spanning 4-domains subfamily A member 4A-like	100911403	4.03
Ms4a6c	Membrane-spanning 4-domains, subfamily A, member 6C	690930	2.76
Anxa2	Annexin A2	56611	2.74
LOC290595	Hypothetical gene supported by AF152002	290595	2.73
Ms4a4a	Membrane-spanning 4-domains, subfamily A, member 4A	361734	2.61
CD206	Mannose receptor, C type 1	291327	2.58
Lpl	Lipoprotein lipase	24539	2.48
Ccl6	Chemokine (C-C motif) ligand 6	287910	2.42
Anxa5	Annexin A5	25673	2.28
Sdc4	Syndecan 4	24771	2.24
Stom	Stomatin	296655	2.19
Rasgef1b	RasGEF domain family, member 1B	100361238	2.19
Cd8b	CD8b molecule	24931	2.18
Gpnmb	Glycoprotein nmb	113955	2.09
Tnfsf9	Tumor necrosis factor superfamily member 9	353218	2.09
Atp6v1e1	ATPase H+ transporting V1 subunit E1	297566	2.06
Rab32	RAB32, member RAS oncogene family	365042	2.05
Mfsd1	Major facilitator superfamily domain containing 1	361957	2.01
Slirp	SRA stem-loop interacting RNA binding protein	688717	1.99
Igf-1	Insulin-like growth factor 1	24482	1.99
Pygl	Liver glycogen phosphorylase	64035	1.98
Igsf6	Immunoglobulin superfamily, member 6	171064	1.98
Enpp6	Ectonucleotide pyrophosphatase/phosphodiesterase6	306460	1.95
Tnfaip2	TNF alpha induced protein 2	299339	1.95

The 25 most upregulated genes in ipsilateral SVZ microglia after HI compared to sham SVZ microglia at P10 (FDR < 0.05)

**Table 3:** Upregulated genes in SVZ microglia after HI at P20

Gene symbol	Gene name	EntrezID	FC HI vs. sham
LOC100910270	Uncharacterized	100910270	6.37
Slfn4	Schlafen 4	114247	4.51
Plac8	Placenta-specific 8	360914	4.12
RT1-Da	RT1 class II, locus Da	294269	3.06
RGD1563091	Similar to OEF2	500011	2.59
Clec7a	C-type lectin domain family 7, member A	502902	2.57
Rsad2	Radical S-adenosyl methionine domain containing 2	65190	2.54
Clec12a	C-type lectin domain family 12, member A	680338	2.51
Ifi27	Interferon, alpha-inducible protein 27	170512	2.45
Tlr1	Toll-like receptor 1	305354	2.26
Pdpn	Podoplanin	54320	2.18
Eif2ak2	Eukaryotic translation initiation factor 2-alpha kinase 2	54287	2.17
Stat1	Signal transducer and activator of transcription 1	25124	2.17
LOC102553917	Putative zinc finger protein 724-like	102553917	2.16
Ifi27l2b	Interferon, alpha-inducible protein 27 like 2B	299269	2.16
RT1-Ba	RT1 class II, locus Ba	309621	2.14
F10	Coagulation factor X	29243	2.14
ND3	NADH dehydrogenase subunit 3	26199	2.12
LOC100911190	Probable ATP-dependent RNA helicase DDX60-like	100911190	2.11
Oas1b	2-5 oligoadenylate synthetase 1B	246268	2.09
C3	Complement C3	24232	2.07
LOC679827	Similar to Ran-specific GTPase-activating protein	679827	2.06
Mir146a	microRNA 146a	100314241	2.06
Cxcl17	C-X-C motif chemokine ligand 17	308436	2.05
Mx2	MX dynamin like GTPase 2	286918	2.02

The 25 most upregulated genes in ipsilateral SVZ microglia after HI compared to sham SVZ microglia at P20 (FDR < 0.05)

### SVZ microglia do not polarize to a M1/M2 state after HI but enrich pathways of neurodegenerative diseases

We examined whether after HI, SVZ microglia express markers associated with the M1 or M2 polarization state. An analysis based on mined microarray data [26] did not support a clear distinction at any given time point (Fig. 5d). We then performed a KEGG pathway analysis of differentially expressed genes of HI and sham SVZ microglia (Fig. 5e). This analysis revealed in HI SVZ microglia an enrichment of pathways associated with neurodegenerative diseases, including oxidative phosphorylation, ribosome, Alzheimer’s (AD), Parkinson’s, and Huntington’s disease. Interestingly, these pathways were also enriched in microglial transcriptome studies in murine models for AD [32] and amyotrophic lateral sclerosis (ALS) [33]. A comparative analysis had previously identified a consensus microglial transcriptional profile among different models of neurodegenerative diseases that was distinct from a LPS-stimulated inflammatory microglial response [32]. Multiple genes of this consensus profile, including Anxa2, Anxa5, Gpnmb, Mfsd1, Igf-1, Clec7, Rsad2, Eif2ak2, Stat1, and C3, were upregulated in SVZ microglia compared to CX microglia with emphasis on HI microglia at P10 (Additional file 1: Figure S7).

### Microglial depletion reduces neurosphere formation

Neurospheres derived from the neonatal SVZ after HI grow in higher numbers than those from sham SVZ, reflecting the HI-induced neurogenesis [28]. By conditional depletion, we explored if microglia affect this HI-induced neurosphere formation. We focused on the P10 time point where the HI-mediated effects on SVZ microglia were most striking.

**Fig. 6:**
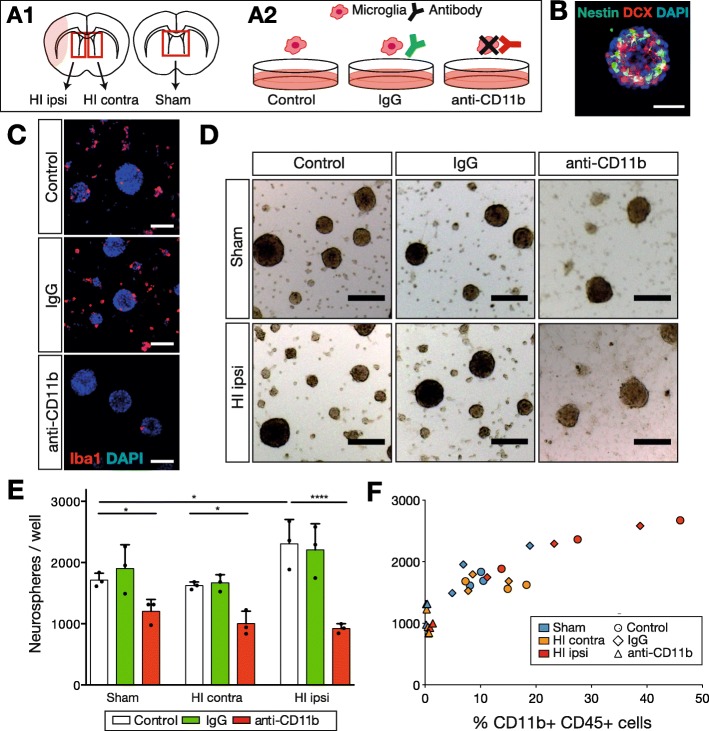
Microglia depletion reduced neurosphere growth in vitro. **A1**, **A2** Experimental design for a neurosphere assay with conditional depletion of microglia. **A1** Tissue origin: ipsi- or contralateral tissue blocks including the SVZ were separately isolated from P10 HI animals (HI ipsi, HI contra), or pooled from P10 sham animals (sham). **A2** Depletion method: dissociated tissue blocks were exposed to three conditions, with either no antibody (control), addition of nonspecific toxic antibodies (IgG), or CD11b+ specific toxic antibodies (anti-CD11b). **B** Representative image of a Nestin+ DCX+ neurosphere after 6 DIC. **C** Representative images of Iba1+ microglia in HI ipsi cell cultures after 6 DIC. The anti-CD11b condition strikingly reduced Iba1+ cells. **D** Representative images from sham and HI ipsi cell cultures after 6 DIC. Less neurospheres were present in the anti-CD11b conditions. **E** Number of neurospheres after 6 DIC. In the control and IgG conditions, HI ipsi-derived tissue cultures produced more neurospheres than sham-derived, reflecting HI-induced proliferation and indicating no interference of unspecific toxic antibodies with cell growth. In contrast, anti-CD11b antibodies significantly decreased neurosphere numbers independent of tissue origin. **F** Data from **E** in comparison to microglial percentage at DIC6, showing a positive correlation between microglial proportions and neurospheres (*r* = 0.87, 95% CI 0.68–0.93). Individual data shown as dots; bars as mean with SD (error bar). One-way ANOVA with Holm-Sidak post hoc test, *p < 0.05, ****p < 0.0001 (Additional file 1: Table S3). Scale bar for **B** 50 μm, **C** 100 μm, **D** 200 μm

The SVZ from P10 HI or sham animals were microdissected and pooled in three tissue groups (Fig. 6 A1): (i) ipsilateral HI, (ii) contralateral HI, and (iii) sham. Cell cultures from each tissue group were subjected to three different conditions (Fig. 6A2): (a) control (no microglial depletion), (b) IgG (addition of a non-specific saporin-conjugated antibody), or (c) anti-CD11b (addition of a CD11b-specific saporin-conjugated antibody). After 6 DIC, neurospheres were formed and expressed nestin and DCX (Fig. 6B). At this stage, CD11b+ CD45+ microglia from all tissue groups were highly depleted in the anti-CD11b culture condition (mean 98% reduction) when measured with a flow cytometer (Additional file 1: Table S2). Immunocytochemical stainings confirmed these results with microglia abundantly attached as single cells or part of neurospheres in the control and IgG conditions, and only few microglia detectable within the neurospheres in the anti-CD11b condition (Fig. 6C). Thus, microglia were an abundant part of the neurosphere culture and were effectively depleted by the anti-CD11b antibody condition.

Next, we quantified the number of neurospheres (Fig. 6D, E). In the control condition, neurosphere numbers were increased in ipsilateral HI compared to sham tissue cultures (ipsilateral HI, 2308 ± 363 vs. sham, 1714 ± 193 neurospheres/well, *p* < 0.027). The IgG condition resulted in similar numbers as the control culture condition among all tissue groups, indicating that the non-specific saporin-conjugated antibody did not impair neurosphere formation. Strikingly, treatment with the anti-CD11b antibody led to a significant decrease in neurosphere numbers in all tissue groups, when compared to control culture conditions (*p* < 0.027). Furthermore, we noted a positive correlation between CD11b+ CD45+ microglial cell fractions and neurosphere numbers (Spearman coefficient *r* = 0.87, 95% CI 0.68–0.93, *p* < 0.0001, *n* = 27) among all experiments (Fig. 6F).

In summary, these findings indicated that CD11b+ CD45+ SVZ microglia supported neurosphere generation in vitro in a concentration-dependent manner. Cultures with ipsilateral SVZ microglia resulted in the highest number of neurospheres while microglia depletion significantly reduced them.

## Discussion

Microglia in the SVZ are increasingly recognized as modulators of neurogenesis [9] and undergo crucial developmental stages in the early postnatal rodent brain [16–20, 22]. In this study, we characterized the phenotype of microglia in the SVZ and adjacent brain regions during early development in rats and investigated the impact of a neonatal HI injury. As HI injury severity bears a considerable variability, only animals with mild to moderate injury were included in our analysis. This allowed to describe a relatively homogeneous experimental cohort. While exact calculation of HI injury size was available for the histological experiments, severity grading for the transcriptomic and cell culture experiments were based on microscopical assessment during dissection, thus reflecting a limitation for a direct comparison of HI injury severity among the different experiments. Our results point to a SVZ-specific response of microglia with a complex gene expression pattern revealing both immunomodulatory and potential neurotrophic effects on neurogenesis.

### Sustained early accumulation and prolonged activation of SVZ microglia after HI

In comparison with adjacent brain regions, early postnatal SVZ microglia showed a region-specific early accumulation and prolonged activation after HI. Furthermore, a corresponding accumulation of proliferating NPCs within the SVZ could not be observed, suggesting that the microglial features are cell type-specific. Similar findings had been previously reported in the adult rat after 2 h of middle cerebral artery occlusion (MCAO) [10], although with major differences. In particular, the SVZ microglial response lasted over 16 weeks in the adult rat and was limited to the ipsilateral SVZ. In comparison, the response in the neonate evolved mainly within 4 weeks and included the contralateral SVZ that was evoked by the combination of hypoxia and ipsilateral ischemic injury, but not by hypoxia alone. An increased neurogenesis in contralateral SVZ has been noted previously after neonatal HI [6, 7]. Several potential factors may account for these differences including the injury model, sex distribution, rat strains, and the cell counting method, which in our study considered changes in SVZ size. Despite these and probably more factors, we propose that a major factor between the two studies is the developmental stage of the SVZ microglia which leads to a different time course of accumulation and activation of early postnatal and adult microglia. Notably, a study with 30 min MCAO was not able to reproduce the aforementioned findings in the adult rat [34]. This suggests that stroke severity affects the adult SVZ microglial response. In our study, we found no correlation between the mild to moderate HI injury size and the SVZ microglial density and activation status (data not shown).

### Increased SVZ microglial proliferation and phagocytosis after HI

BrdU-labeling indicated an early endogenous SVZ microglial proliferation that is potentiated by HI injury. Whether this additional proliferation was solely responsible for the sustained early accumulation observed after HI or to which extent migrating myeloid cells contributed cannot be fully answered in this study. The gene expression analysis of CD11b+ SVZ cells showed high expression of signature microglial genes, thereby pointing out that the vast majority of these cells were of microglial origin.

In sham animals, the proportion of amoeboid microglia in the SVZ decreased continuously with age. HI injury temporarily increased this proportion in both the ipsilateral and contralateral SVZ before returning to levels similar to those in sham animals.

To our knowledge, microglial phagocytosis has not been quantified in the early postnatal SVZ. Microglial phagocytosis is fundamental for CNS development and maintenance, immune defense, and tissue repair. In mature ramified microglia, ball-and-chain-shaped phagocytic buds that engulf apoptotic cells from the proximity have been described [12, 29, 30, 35]. It is possible that ball-and-chain buds in ramified early postnatal microglia are also of the same phagocytic nature, although in contrast to the adult, they were not systematically demonstrating engulfement of cells that expressed markers for apoptosis or typical antigens for other SVZ cells. During physiological development, the proportion of ball-and-chain buds dramatically declined between P10 and P40, while it increased during the same period in HI-exposed animals. Of note, ball-and-chain phagocytic terminal branches are best recognized in ramified microglia which are less present at earlier time points. Therefore, this analysis most likely underestimates the overall phagocytic activity at earlier time points, especially after HI, since non-ramified microglia are highly phagocytic [22]. It is speculated that the increased proportion of ball-and-chain buds after HI impacts the neurogenic niche, especially since a marked proportion of engulfed cells had recently proliferated. Even though the phagocytic nature of these microglial ball-and-chain protrusions is not fully ascertained, their increased proportion at P40 underlines the long-term effects of HI on SVZ microglia.

### Gene expression analysis indicates spatially and temporarily distinct patterns in SVZ microglia

We performed a microarray transcriptome analysis of CD11b+ microglia isolated from SVZ and cortex of individual sham and HI animals. Our results indicated a high purity of CD11b+ cells expressing microglial signature genes. In this analysis, neuroectodermal genes, and among them most prominently DCX, were also expressed by microglia, as previously reported [17, 36, 37]. Whether this reflects a true microglial gene expression or the uptake of mRNA from neighboring cells remains to be established [37].

The microglial transcriptome showed significant differences between anatomical regions. Among the most upregulated genes in the SVZ were those recently described in pooled whole-brain transcriptome studies of early postnatal murine microglia [17, 19, 20]. Our data from individual rats add to these findings by identifying the SVZ microglia as a specific source for these gene patterns. However, the microarray data could not identify a CD11c+ subpopulation in the rat SVZ as previously reported in mice [17].

The gene enrichment analysis resulted in a clear difference between sham and HI SVZ microglia. Interestingly, categories concerning CNS development were present in sham animals at P10, and again, in HI animals at P20, suggesting common functional properties.

After HI, SVZ microglia upregulated both pro- and anti-inflammatory genes until P20. Importantly, microglial markers of acute inflammation such as IL-1β, IL-6, TNF-ɑ, and IFN- γ[38–40] were not differentially expressed (data not shown), underscoring that the SVZ microglial response was different to that seen in microglia nearby ischemic tissue. Among the most upregulated genes in P10 SVZ microglia after HI were neurotrophic genes, such as IGF-1 and Gpnmb. Microglia-derived IGF-1 is essential for cortical neuronal survival [15], is neuroprotective after ischemic stroke [41], and is upregulated in the adult rat SVZ and striatal microglia after ischemic stroke [10, 34]. Gpnmb, also known as Osteoactivin, is predominantly expressed by microglia in the rodent brain [42, 43], especially in the early postnatal stage [17, 19]. The role of Gpnmb in rat microglia is not fully understood. In mice, it shows anti-inflammatory properties in macrophages [44] and neuroprotective effects in models of brain ischemia [45] and ALS [46]. In conclusion, these results point out to a spatially and temporally distinct gene expression pattern of SVZ microglia, which after HI is outlined by pro- and anti-inflammatory as well as neurotrophic genes.

### SVZ microglia display pathways associated with neurodegenerative diseases after HI

The M1 and M2 polarization state of microglia has recently been questioned by several in vivo microglial transcriptome studies [32, 33, 42, 47, 48]. Even though some characteristic polarization markers, such as CD206 or Stat1, were found among the most upregulated genes in HI-exposed SVZ microglia, the analysis did not indicate a clear trend to adopt a polarization state after neonatal HI.

The KEGG pathway enrichment analysis in SVZ microglia after HI pointed to pathways associated with neurodegenerative diseases. Interestingly, similar findings were reported in several microglia studies in mouse models for ALS, AD, accelerated, and physiological aging that were distinct from a microglial response to LPS exposure [32, 33, 49]. From these studies, a microglial consensus gene set, including Igf-1 and Gpnmb, was identified [32]. When applied to our study, this consensus gene set was mostly expressed in SVZ microglia at P10 with emphasis on HI-treated animals. This result was unexpected, and despite several methodological differences, it can be speculated that it may partially reflect a microglial state shared by radically distinct CNS pathologies [48, 50].

### SVZ microglia support neurosphere generation in vitro

HI-induced proliferation of NSC and NPC can be recapitulated in an in vitro neurosphere assay. By specific depletion, we assessed the effect of SVZ microglia on neurosphere formation. In line with previous reports [28], cultures from ipsilateral HI SVZ display higher numbers of neurospheres than those from sham SVZ. Strikingly, this was completely abolished when microglia were depleted. Microglial percentage and neurosphere numbers in culture positively correlated among all experimental conditions, suggesting that microglia support neurosphere formation in a concentration-dependent manner.

These results are in line with previous in vitro studies demonstrating trophic effects of microglia. Conditioned medium from mixed cell cultures containing microglia from young and ischemic rodent brain tissue promoted NPC proliferation [51, 52]. In co-culture systems, microglia had inhibitory effects on NPC differentiation and attenuated negative effects of LPS or IFN-γ on murine neurosphere formation [53, 54]. Also, secreted compounds stimulated neurosphere proliferation like IL-6, most likely derived from microglia [55], and IGF-1 [56], which we found to be specifically and highly expressed by SVZ microglia after HI. SVZ-derived NPCs establish functional gap junctions with microglia [57]. In our in vitro experiments, microglia were an integral part of neurospheres. Hence, it is possible that microglia modulate NPC not only via secreted factors but also through direct cell-cell interaction. Furthermore, the interaction between microglia and NPC is reciprocal with NPC enhancing microglial proliferation [53, 58], which is underlined by our results, where microglial proportions positively correlated with neurosphere numbers with the highest values in cultures from the ipsilateral HI SVZ. Future studies are warranted to identify the SVZ microglial mediators which enhance neurosphere formation after HI.

## Conclusions

This report provides an in-depth characterization of the microglial phenotype in the rat SVZ after neonatal HI. In summary, microglia in the early postnatal SVZ are a distinct microglial sub-type in terms of morphology and gene expression profile. HI injury has lasting effects on their developmental course, as shown by their cellular accumulation, prolonged activation, and sustained phagocytosis, which do not occur in adjacent brain regions. After HI, SVZ microglia upregulate a heterogeneous pattern of genes related to pro- and anti-inflammatory as well as neurotrophic effects. This transcriptomic profile does not strictly reflect a M1 or M2 polarized state, but coincides to some extent with recently described microglial gene expression patterns found in several models of neurodegenerative diseases. Early postnatal microglia correlate with neurosphere generation in vitro in a concentration-dependent manner, suggesting a potentially reciprocal trophic effect.

HIE still bears a high risk for life-long sequelae for the affected patients. Thus, there is a need for therapies aiming at enhancing endogenous repair mechanisms. To evaluate such new therapeutic approaches, a better understanding of the cellular players in the pathomechanisms is necessary. Concurrent beneficial and detrimental effects mediated through microglia after CNS damage underscore the versatility, and thus the difficulty for their characterization. This study provides new insight into the specific response of early postnatal SVZ microglia upon HI injury. Our findings suggest that SVZ microglia might play a beneficial role for neurogenesis which merits further research. Overall, this study highlights the significance of the microenvironment and of the developmental stage on the microglial phenotype.

## Supplementary information

**Additional file 1: Table S1.** Total numbers of animals, numbers of included animals with sex distribution and excluded animals with reason for exclusion for each experiment.** Table S2.** Percentages of CD11b+ CD45+ microglia per tissue group and condition for each experiment. **Table S3.** List of all statistical comparisons in the main figures. **Figure S1.** Right and left hemispheres in sham animals are not different. **Figure S2.** Density of PCNA+ and PAX6+ cells in the dorsolateral SVZ. **Figure S3.** Hypoxia alone is insufficient to elicit HI-specific microglial changes. **Figure S4.** SVZ microglial phagocytosis of Ki67+ SVZ cells. **Figure S5.** Magnetic bead sorting resulted in a high purity of CD11b+ CD45+ microglia. **Figure S6.** Microarray validation with qPCR for Igf-1. **Figure S7.** The gene expression profile of cortex and SVZ microglia after neonatal HI shared similarities with that of microglia from rodent models of neurodegenerative diseases.

## Data Availability

The microarray datasets generated and analyzed during the current study are available in the GEO gene expression omnibus repository (GSE97299). The other datasets used and analyzed during the current study are available from the corresponding author on reasonable request.

## Abbreviations

AD: Alzheimer’s disease
ALS: Amyotrophic lateral sclerosis
BRDU: Bromodeoxyuridine
CC: Corpus callosum
CC3: Cleaved caspase 3
CNS: Central nervous system
CX: Cortex
DCX: Doublecortin
DIC: Day in culture
FC: Fold change
FDR: False detection rate
GO: Gene ontology
HBSS: Hank’s balanced salt solution
HI: Hypoxia-ischemia
HIE: Hypoxic-ischemic encephalopathy
Iba1: Ionized calcium-binding adapter molecule 1
KEGG: Kyoto Encyclopedia of Genes and Genomes
LPS: Lipopolysaccharide
MCAO: Middle cerebral artery stroke
NPC: Neural progenitor cell
NSC: Neural stem cell
P: Postnatal day
PAX6: Paired box 6 protein
PB: Phosphate buffer
PCNA: Proliferating nuclear cell antigen
PFA: Paraformaldehyde
PSA-NCAM: Polysialylated-neural cell adhesion molecule
qPCR: Quantitative real-time polymerase chain reaction
RT: Room temperature
SD: Standard deviation
SOX2: Sex-determining region Y-box 2
SVZ: Subventricular zone
TBS: Tris-buffered saline
