# Supplementary material for: Neonatal hypoxia-ischemia in rat elicits a region-specific neurotrophic response in SVZ microglia
Source: J Neuroinflammation. 2020 Jan 18;17:26. doi: 10.1186/s12974-020-1706-y (PMC6969423; doi:10.1186/s12974-020-1706-y)
Supplement: Supplementary file 1 — Additional file 1: Table S1. Total numbers of animals, numbers of included animals with sex distribution and excluded animals with reason for exclusion for each experiment. Table S2. Percentages of CD11b+ CD45+ microglia per tissue group and condition for each experiment. Table S3. List of all statistical comparisons in the main figures. Figure S1. Right and left hemispheres in sham animals are not different. Figure S2. Density of PCNA+ and PAX6+ cells in the dorsolateral SVZ. Figure S3. Hypoxia alone is insufficient to elicit HI-specific microglial changes. Figure S4. SVZ microglial phagocytosis of Ki67+ SVZ cells. Figure S5. Magnetic bead sorting resulted in a high purity of CD11b+ CD45+ microglia. Figure S6. Microarray validation with qPCR for Igf-1. Figure S7. The gene expression profile of cortex and SVZ microglia after neonatal HI shared similarities with that of microglia from rodent models of neurodegenerative diseases. [file 12974_2020_1706_MOESM1_ESM.pdf]

Supplementum:

Neonatal Hypoxia-Ischemia in rat elicits a region-specific neurotrophic response in SVZ Microglia

Urs Fisch[1,2*], Catherine Brégère[2], Florian Geier[3,4], Laurie Chicha[2], Raphael Guzman[2,5,6]

1. Department of Neurology, University Hospital Basel, University Basel, Basel, Switzerland
2. Brain ischemia and regeneration, Department of Biomedicine, University Hospital Basel, University Basel, Basel, Switzerland
3. Bioinformatics Core Facility, Department of Biomedicine, University Basel, Basel, Switzerland
4. Swiss Institute of Bioinformatics, Basel, Switzerland
5. Department of Neurosurgery, University Hospital Basel, University Basel, Basel, Switzerland
6. Faculty of Medicine, University Basel, Basel, Switzerland

Index:

A) Tables

Page 2:

Supplemental table 1: Total numbers of animals, numbers of included animals with sex distribution and excluded animals with reason for exclusion for each experiment.

Supplemental table 2: Percentages of CD11b+ CD45+ microglia per tissue group and condition for each experiment.

Page 3:

Supplemental table 3: List of all statistical analysis in the main figures.

B) Figures

Page 3:

Supplemental figure 1: Right and left hemispheres in sham animals are not different.

Page 4:

Supplemental figure 2: Density of PCNA+ and PAX6+ cells in the dorsolateral SVZ.

Supplemental Figure 3: Hypoxia alone is insufficient to elicit HI-specific microglial changes.

Supplemental Figure 4: SVZ microglial phagocytosis of Ki67+ SVZ cells.

Page 5:

Supplemental Figure 5: Magnetic bead sorting resulted in a high purity of CD11b+ CD45+ microglia.

Supplemental Figure 6: Microarray validation with qPCR for Igf-1.

Supplemental Figure 7: The gene expression profile of cortex and SVZ microglia after neonatal HI shared similarities with that of microglia from rodent models of neurodegenerative diseases.

A) Tables:

Experiment	Age	Surgical group	Total number of animals	Included animals (female:male)	Excluded animals (reason)
**Histology**	P10	HI	7	5 (3:2)	2 (HI injury severity)
**Histology**	P10	Sham	5	5 (2:3)	0
**Histology**	P20	HI	7	5 (3:2)	2 (HI injury severity)
**Histology**	P20	Sham	5	5 (3:2)	0
**Histology**	P40	HI	8	5 (3:2)	3 (HI injury severity)
**Histology**	P40	Sham	5	5 (3:2)	0
**Histology**	P10	Hypoxia only	3	3 (2:1)	0
**Microarray**	P10	HI	24	6 (2:4)	18 (12 HI injury severity, 6 insufficient RNA extraction)
**Microarray**	P10	Sham	11	6 (2:4)	5 (insufficient RNA extraction)
**Microarray**	P20	HI	20	6 (3:3)	14 (11 HI injury severity, 3 insufficient RNA extraction)
**Microarray**	P20	Sham	7	6* (4:2)	1 (insufficient RNA extraction)
**qPCR**	P10	HI	2	2 (1:1)	0
**qPCR**	P10	Sham	2	2 (1:1)	0
**qPCR**	P20	HI	2	2 (1:1)	0
**qPCR**	P20	Sham	2	2 (1:1)	0
**Cell culture**	P10	HI	22	18 (10:8)	4 (HI injury severity)
**Cell culture**	P10	Sham	9	9 (5:4)	0

Supplemental table 1: Total numbers of animals, numbers of included animals with sex distribution and excluded animals with reason for exclusion for each experiment. *During microarray normalization steps, one animal was identified as an outlier and removed from further analysis (see main text).

		Day 0 at seeding<br>CD11b+ CD45+ cells (%)			Day in culture 6<br>CD11b+ CD45+ cells (%)			Relative reduction of CD11b+ CD45+ cells from seeding to Day in culture 6<br>CD11b+ CD45+ cells (%)			
Tissue group	Condition	Experiment 1	Experiment 2	Experiment 3	Experiment 1	Experiment 2	Experiment 3	Experiment 1	Experiment 2	Experiment 3	**Average**
Sham	Control	29.0	13.7	25.0	10.5	8.1	10.1	63.8	40.9	59.6	**54.8**
	IgG				18.9	4.9	6.9	34.8	64.2	72.4	**57.2**
	anti-CD11b				0.2	0.2	0.4	99.3	98.5	98.4	**98.8**
HI contra-lateral	Control	41.6	16.0	24.5	14.9	18.3	7.3	64.2	-14.4	70.2	**40.0**
	IgG				15.1	8.6	7.8	63.7	46.3	68.2	**59.4**
	anti-CD11b				0.5	0.3	0.6	98.8	98.1	97.6	**98.2**
HI ipsi-lateral	Control	42.6	31.6	34.9	46.0	27.5	13.8	-8.0	13.0	60.5	**21.8**
	IgG				38.8	23.3	11.2	8.9	26.3	67.9	**34.4**
	anti-CD11b				0.9	1.4	0.7	97.9	95.6	98.0	**97.2**

Supplemental table 2: Percentages of CD11b+ CD45+ microglia per tissue group and condition for each experiment. The relative reduction of CD11b+ CD45+ cells were similar between the control and IgG condition. However, the anti-CD11b condition efficiently depleted these cells. The relative reduction of CD11b+ CD45+ cells was calculated as the relative difference (%) between CD11b+ CD45+ cells % $_{seeding}$ and CD11b+ CD45+ cells % $_{Day\ 6}$.

Analysis	Comparison of experimental groups	(adjusted) p-value
SVZ size (Fig. 1A)	P10 sham vs P10 HI ipsi	0.0034
	P10 HI ipsi vs P20 HI ipsi	0.0005
	P10 HI ipsi vs P40 HI ipsi	< 0.0001
Microglia density (Fig. 2C)	SVZ P10 sham vs SVZ P10 HI contra	0.0037
	SVZ P10 sham vs SVZ P10 HI ipsi	< 0.0001
	SVZ P20 sham vs SVZ P20 HI ipsi	0.0007
	SVZ P40 sham vs SVZ P40 HI ipsi	< 0.0001
	SVZ P40 HI contra vs SVZ P40 HI ipsi	0.002

	CC P10 sham midline vs CC P10 HI midline	< 0.0001
	CC P10 HI midline vs CC P40 HI midline	< 0.0001
Proportion of activated microglia (Fig. 2D)	SVZ P10 sham vs SVZ P10 HI contra	0.009
	SVZ P10 sham vs SVZ P10 HI ipsi	<0.0001
	SVZ P20 sham vs SVZ P20 HI ipsi	0.0058
	SVZ P40 sham vs SVZ P40 HI ipsi	0.0822
	SVZ P10 sham vs SVZ P20 sham	0.009
	SVZ P10 sham vs SVZ P40 sham	0.001
	SVZ P10 HI ipsi vs SVZ P20 HI ipsi	< 0.0001
	SVZ P10 HI ipsi vs SVZ P40 HI ipsi	< 0.0001
	CX P10 sham vs CX P10 HI ipsi	0.0028
	CX P10 HI ipsi vs CX P40 HI ipsi	0.0022
	CC P10 sham midline vs CC P10 HI midline	0.0014
	CC P10 HI midline vs CC P40 HI midline	< 0.0001
Microglial proliferation (Fig. 3)	SVZ P10 sham vs SVZ P10 HI contra	0.0015
	SVZ P10 sham vs SVZ P10 HI ipsi	0.0136
Proportion of microglia with ramified morphology (Fig. 4B)	P10 sham vs P10 HI contra	0.0009
	P10 sham vs P10 HI ipsi	0.001
	P10 HI vs P20 HI ipsi	0.0017
	P10 HI vs P40 HI ipsi	< 0.0001
Microglial ball-and-chain phagocytosis (Fig. 4D)	SVZ P10 sham vs SVZ P10 HI contra	0.0006
	SVZ P10 sham vs SVZ P10 HI ipsi	< 0.0001
	SVZ P40 sham vs SVZ P40 HI contra	0.0011
	SVZ P40 sham vs SVZ P40 HI ipsi	0.0083
	SVZ P10 sham vs SVZ P40 sham	0.0002
	SVZ P10 HI ipsi vs SVZ P40 HI ipsi	0.0026
Neurosphere culture with conditional microglial depletion (Fig. 6E)	sham control vs sham anti-CD11b	0.0268
	HI contra control vs HI contra anti-CD11b	0.0267
	HI ipsi control vs HI ipsi anti-CD11b	< 0.0001
	sham control vs HI ipsi control	0.0267

Supplemental table 3: List of all statistical analysis in the main figures. See main text for abbreviations and details.

B) Figures:

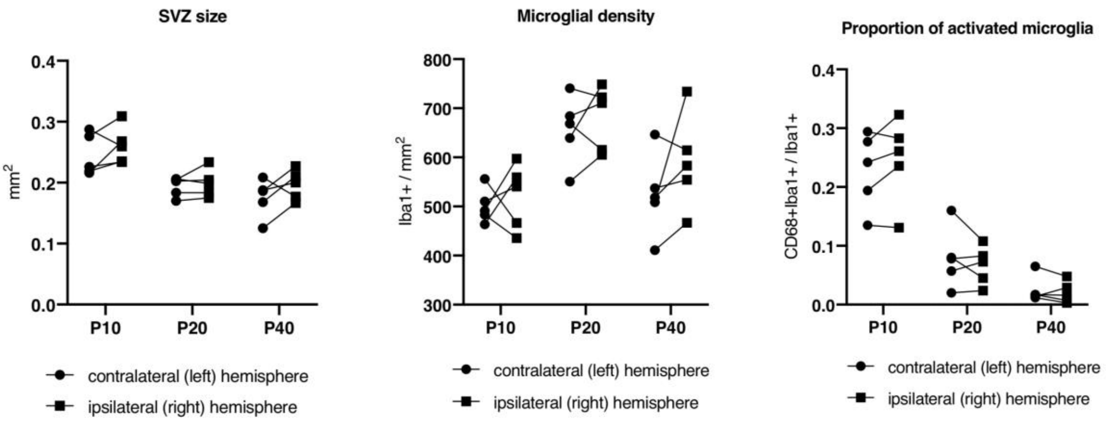

Supplemental figure 1: Right and left hemispheres in sham animals are not different. A paired comparison of the ipsilateral (right) and contralateral (left) hemisphere in individual animals after ipsilateral sham surgery indicated no differences between the two hemispheres (Paired two-tailed t-test for SVZ size P10 p=0.30, P20 p=0.41, P40 p=0.21; for microglial density P10 p=0.65, P20 p=0.45, P40 p=0.20; for proportion of activated microglia P10 p=0.19, P20 p=0.40, *P40* p=0.47; n=5 animals per time point).

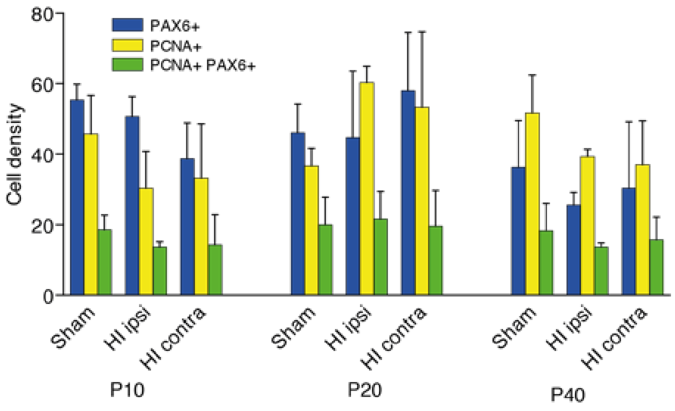

Supplemental figure 2: Density of PCNA+ and PAX6+ cells in the dorsolateral SVZ. Due to the vast cellular density and extensive neurogenesis in the early postnatal SVZ, NPC were identified by the nuclear marker PAX6 for a most accurate cell counting. The densities of PCNA+, PAX6+ or PCNA+ PAX6+ cells in the SVZ were not markedly different between HI and sham animals at any time point. Bars as mean with SD (error bars), n=3 animals for sham, n=3 animals for HI per time point.

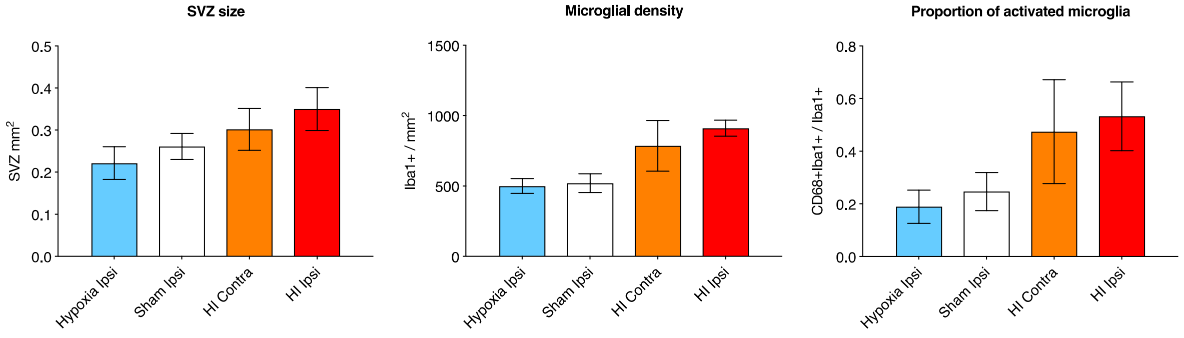

Supplemental figure 3: Hypoxia alone is insufficient to elicit HI-specific microglial changes. P10 animals exposed to global hypoxia at P7 without occlusion of the common carotid artery did not show any difference in SVZ size, microglial density or proportion of activated microglia, compared to age-matched animals with sham or HI surgery at P7. This finding indicates that hypoxia alone is not sufficient to elicit microglial changes observed in both the ipsilateral and contralateral SVZ after HI. Mean with SD (error bars), n=3 animals for hypoxia, n=5 animals for sham, n=5 animals for HI.

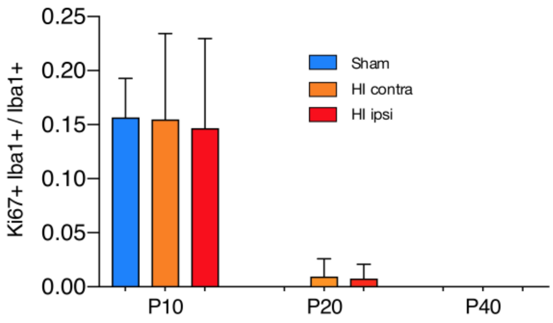

Supplemental Figure 4: SVZ microglial phagocytosis of Ki67+ SVZ cells. At P10, a considerable number of microglial ball-and-chain engulfed nuclei stained positive for Ki67 without any difference between HI or sham surgery. At P20, they were very rarely seen in the HI SVZ, and were absent at P40. Mean with SD (error bar), n=3 animals for sham, n=3 animals for HI per time point.

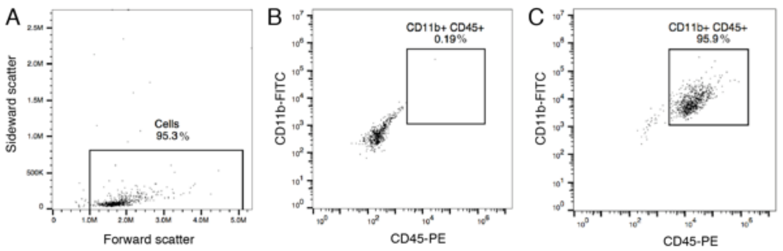

Supplemental Figure 5 (above): Magnetic bead sorting resulted in a high purity of CD11b+ CD45+ microglia. Cortical microglia isolated by CD11b+ magnetic bead sorting. (A) Gating strategy for cell identification. (B) Unstained isolated cortical cells. (C) After staining with the corresponding antibodies, isolated cortical cells were >95% CD11b+ CD45+.

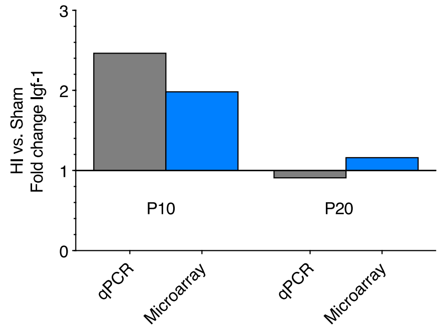

Supplemental Figure 6 (above): Microarray validation with qPCR for Igf-1. The microarray data (Table 2, 3) were validated by qPCR using SVZ samples from additional animals that were not previously used in the microarray analysis. Due to the very low concentration of sample RNA, validation was performed for Igf-1, which was significantly differentially expressed at P10, but not anymore at P20. The Igf-1 fold change measured by qPCR of additional samples were in line with the fold change by microarray analysis (n=2 animals for sham, n=2 for HI per time point).

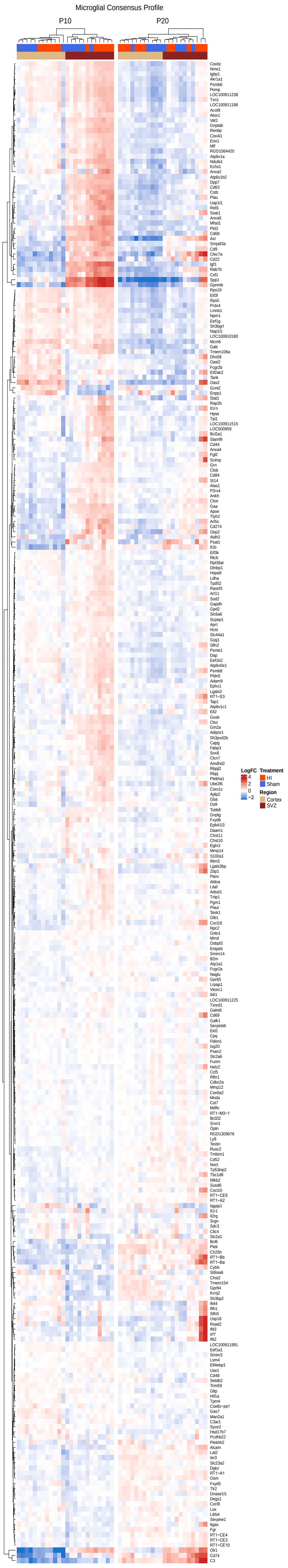

Supplemental Figure 7 (left): The gene expression profile of cortex and SVZ microglia after neonatal HI shared similarities with that of microglia from rodent models of neurodegenerative diseases. Heat map for a consensus profile of microglial responses in the context of neurodegenerative diseases (from Holtman et al., 2015, see main text). A considerable number of consensus genes were upregulated in SVZ microglia at P10 with emphasis on HI treated animals.
